# When to Graft the Incised Plate During TIP Repair? A Suggested Algorithm That may Help in the Decision-Making Process

**DOI:** 10.3389/fped.2018.00326

**Published:** 2018-11-14

**Authors:** Tariq O. Abbas, Joao L. Pippi Salle

**Affiliations:** ^1^Weill Cornell Medicine-Qatar, Ar-Rayyan, Qatar; ^2^Sidra Medical and Research Center, Doha, Qatar; ^3^College of Medicine, Qatar University, Doha, Qatar

**Keywords:** hypospadias, grafted tubularized incised plate, dorsal inlay graft, urethral plate, Ratio

Although more than 300 different techniques for hypospadias repair do exist, successful outcome depends mainly on the surgeon's skills, availability of adequate tissue for urethral reconstruction and choice of the best suitable technique in each case. Significant advancements in the management of hypospadias occurred over the last few decades but there remains great controversy on how to select the best technical options (1). The general principle for hypospadias repair consists in the tubularization of ventral urethral plate, bringing the meatus all the way to the tip of the glans, depending on the presence of associated ventral penile curvature. Such accomplishment can be achieved with a single or staged procedure. In the most common scenario, distal hypospadias without curvature, tubularization of the urethral plate is the currently most frequently utilized approach (2). However simple tubularization is not always suitable, especially in narrow plates that could result in stenosis. Aiming to augment the plate dimensions Snodgrass, in 1998, popularized to simply deeply incise and tubularize the urethral plate, relying on epithelization/granulation of the raw dorsal urethra (3, 4). This procedure has been successful in a great number of cases, reason why it was quickly adopted worldwide. The mechanism of healing the incised plate is still open for discussion (5). Some authors believe there is complete re-epithelization with urothelium while others think that there is formation of granulation tissue that later is followed by gradual fibrosis of the area. This could be reason why some authors reported worse outcomes in narrow urethral plates, theorizing that most of the neourethra would be reconstructed based on a raw, current dorsal urethrotomy that gradually heals narrowing the segment (4, 6). Moreover, healing of a larger incised raw area of neourethra is unpredictable; it seems that this process may exert tension on the ventral suture line affecting its primary healing (7). In an attempt to improve healing of the neourethra after the TIP urethroplasty, grafting of the dorsal incised (GTIP) area using the inner prepuce has been described by several authors (8–10). It was shown, in a series of 1,095 hypospadias, that there were 0 and 18% complication rate with GTIP in primary and repeat surgeries in contrast to about 5, 23% consecutively (11). Although the GTIP is an attractive and well-accepted procedure by adult reconstructive urologists repairing urethral strictures, its acceptance for primary hypospadias in children is still unclear. GTIP is more technically demanding than regular TIP therefore its indication should be tailored appropriately. Unfortunately, it has been difficult to prove the advantages of the GTIP over the TIP repair as both have low complication rates, demanding a large number of subjects for a randomized study aiming to answer this question. Therefore, at this point in time, we are faced with a procedure that makes sense but still not proved by evidence and most surgeons adopt it based on expert opinion. We have been trying to delineate which patients with hypospadias need to have GTIP, considering those with unfavorable plates (narrow and poor spongiosum) as the ideal candidates. However, based on visual inspection, it is not easy to agree upon urologists what is a favorable or unfavorable plate (12). Considering these difficulties, we would like to propose an objective simple formula, measuring the ratio of the urethra before and after incision, to help deciding to graft or not the incised plate. The urethral plate should be maximally stretched and measured in its widest point. It is then deeply incised all the way to the corpora and the same measurements are repeated, therefore producing a urethral plate ratio before and after incision. If the ratio is less than 0.5 we consider that a significant component of the neourethra will be composed by raw tissue, therefore vulnerable to stenosis and such cases should be grafted (Figure 1).

**Figure 1:**
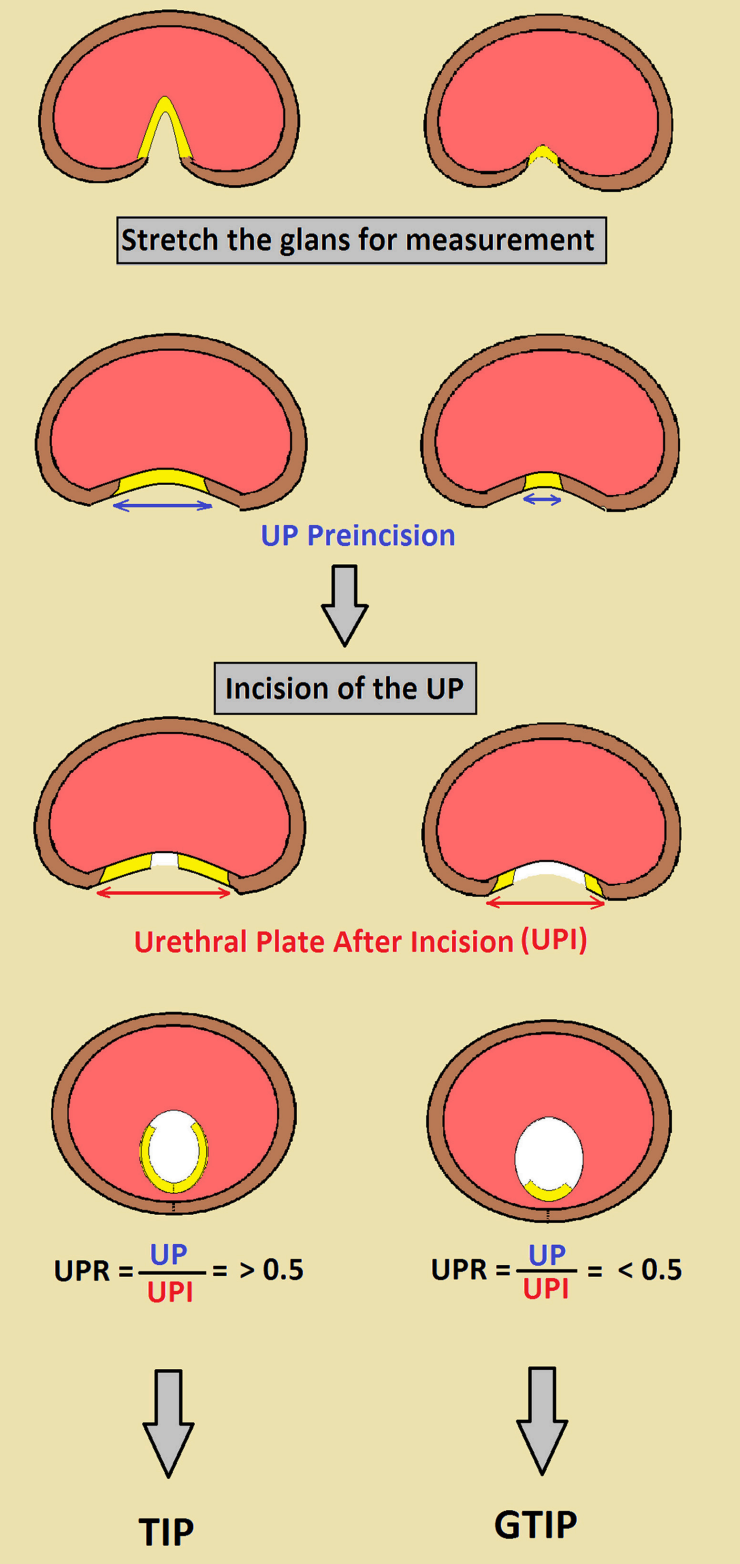
Schematic representation of a cross section at the level of the glans in distal hypospadias repair demonstrating the utilization of the Urethral Plate Ratio.

We acknowledge that such proposal lacks evidence support but it is an objective and consistent measurement that may be useful to surgeons deciding to graft or not the incised plate.

## Author contributions

TA has come up with the suggested UPR ratio. JP has validated the point and both wrote this manuscript.

### Conflict of interest statement

The authors declare that the research was conducted in the absence of any commercial or financial relationships that could be construed as a potential conflict of interest.
